# Prophylactic Mesh Application during Colostomy to Prevent Parastomal Hernia: A Meta-Analysis

**DOI:** 10.1155/2016/1694265

**Published:** 2016-10-12

**Authors:** JunJia Zhu, YuWei Pu, XiaoDong Yang, DeBao Zhang, Kui Zhao, Wei Peng, ChunGen Xing

**Affiliations:** Department of General Surgery, Second Affiliated Hospital, Soochow University, Suzhou, Jiangsu 215004, China

## Abstract

*Background*. Parastomal hernia is a common complication after stoma formation, especially in permanent colostomy. The present meta-analysis aimed to evaluate the effectiveness of prophylactic mesh application during permanent colostomy for preventing parastomal hernia.* Methods.* Randomized controlled trials comparing outcomes in patients who underwent colostomy with or without prophylactic mesh application were identified from PubMed, EMBASE, Science Citation Index, and the Cochrane Libraries.* Results.* This meta-analysis included 8 randomized controlled trials with 522 participants. Our pooled results showed that prophylactic mesh application (mesh group) reduced the incidence of clinically detected parastomal hernia (risk ratio [RR]: 0.22; 95% confidence interval [CI]: 0.13–0.38; *P* < 0.00001), radiologically detected parastomal hernia (RR: 0.62; 95% CI: 0.47–0.82; *P* = 0.0008), and surgical repair for herniation (RR: 0.34; 95% CI: 0.14–0.83; *P* = 0.02) when compared with conventional permanent colostomy formation (control group). The incidence of complications, including wound infection, peristomal infection, mesh infection, stomal necrosis and stenosis, stoma site pain, and fistula, was not higher in the mesh group than in the control group.* Conclusions.* Our meta-analysis demonstrated that prophylactic mesh application at the time of primary colostomy formation is a promising method for the prevention of parastomal herniation.

## 1. Introduction

Parastomal hernia is one of the most frequent colorectal complications noted in patients who have undergone surgical treatment for cancer or inflammatory bowel disease, followed by stoma formation [1]. It is known that colostomy often has an adverse influence on quality of life [2]. The prevalence of parastomal hernia ranges from 5% to 52% in the literature [3–5], and the rate of colostomy-related parastomal hernia has been found to gradually increase with prolonged follow-up time [4, 6].

Although most parastomal hernias remain asymptomatic, many patients complain of pain, stomal appliance problems, skin irritation, and stoma site discomfort [7, 8]. Surgical techniques for repairing parastomal hernias include local fixation, resiting the stoma, and prosthetic mesh repair [9]. However, the recurrence rate remains high after surgical treatment of parastomal hernia [10]. According to a report by Allen-Mersh and Thomson, 47% of patients who have undergone local repair experience hernia recurrence [11]. Prosthetic mesh repair has been recommended by Carne et al., as this approach is associated with low recurrence; however, this technique requires a second surgery and can cause additional complications [12].

Satisfactory techniques for repairing parastomal hernia are lacking, and, therefore, prophylaxis of parastomal hernia is extremely important [12, 13]. Lian et al. reported a low rate of parastomal hernia with extraperitoneal colostomy [14]. Presently, more surgeons are considering prophylactic mesh application at the time of stoma creation. Bayer et al. first described this procedure in 1986 and reported that no parastomal hernia formation was detected in 47 patients [15]. Several previous studies have reported a low rate of hernia formation and a reduced risk of infection with prophylactic mesh application [16–18]. In a previous study by Fleshman et al., there was no difference in the incidence of parastomal herniation between the mesh group and conventional group [19]. However, ileostomy accounted for a large proportion of the procedures along with open and laparoscopic surgery, and maybe this affected the statistical power to detect the differences between two groups. In a report by Carne et al., 4.0–48.1% of colostomies developed parastomal hernias [12]. Considering colostomy is more susceptible to developing the parastomal hernia than ileostomy, the present meta-analysis aimed to evaluate the effectiveness of prophylactic mesh application during permanent colostomy for preventing parastomal hernia.

## 2. Materials and Methods

### 2.1. Search Strategy and Selection Criteria

Multiple databases (PubMed, EMBASE, Science Citation Index, the Cochrane Central Register of Controlled Trials, and Cochrane Register of Systematic Reviews) were searched. The literature search was performed for studies published between January 1980 and April 2016, using the following medical subject headings: “surgical mesh,” “implants,” “enterostomy,” “ostomy,” “surgical stomas,” and “colostomy,” along with free-text words. Two reviewers scanned and evaluated the studies independently.

The inclusion criteria were as follows: (1) comparative trials comparing primary formation of the colostomy with or without prophylactic mesh application; (2) randomized controlled trials (RCTs) published before March 2016; and (3) presence of information on the following outcomes: parastomal hernia, surgery for parastomal hernia, and infectious and noninfectious complications.

### 2.2. Data Collection

Two investigators extracted and documented the relevant information from each study independently, and disagreements were resolved through consultations with a third investigator. If disagreements remained unresolved, the whole study group participated in discussions. The following data were extracted: author, country, year of publication, participant parameters, surgical parameters, sample size, diagnostic method for hernia, and follow-up time. The following parameters were extracted: occurrence of parastomal hernia, incidence of surgery repair for parastomal hernia, and stoma-related and non-stoma-related complications (wound infection, peristomal infection, mesh infection, fistula, stomal necrosis and stenosis, and stomal site pain).

### 2.3. Quality Assessment

We evaluated the methodological quality of the included studies according to the Cochrane Risk of Bias Tool [29]. The assessment included the following 7 items [30]: randomization sequence generation, allocation concealment, blinding of participants and study personnel, blinding of outcome assessors, incomplete outcome data, selective reporting, and other biases. Two investigators resolved disagreements through discussion.

### 2.4. Statistical Analysis

All analyses were performed using the Review Manager software ver. 5.3 (the Nordic Cochrane Centre, Copenhagen, Denmark, 2014). The analyses were performed using risk ratios (RRs) and 95% confidence intervals (CIs) for dichotomous data. A *P* value <0.05 was considered statistically significant.

Heterogeneity was assessed with *I* ^2^ measurement across the studies [31]. Statistical heterogeneity was assessed with *I* ^2^ measurement and was regarded as significant when *I* ^2^ was >50% and *P* value was ≤0.10. A random-effects model was used to combine the data if heterogeneity was present in the results; otherwise, a fixed-effect model was used.

We did not assess publication bias because a small number of trials were included in this meta-analysis.

## 3. Results

### 3.1. Included Studies and Study Characteristics

**Figure 1:**
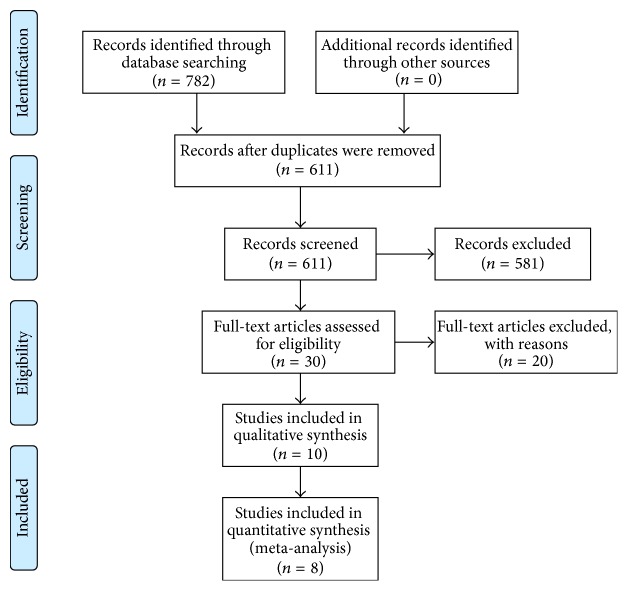
Flow chart of searching strategy for randomized controlled trials.

The search of the electronic databases identified 782 relevant studies. Of these studies, 171 duplicates were excluded. Additionally, 2 investigators screened the title and abstract independently and excluded 581 studies. Thus, 30 studies were finally considered for inclusion. Of these studies, 8 RCTs with 522 participants were finally selected for this meta-analysis [18, 20–28]. The selection procedure has been presented in Figure 1.

**Figure 2:**
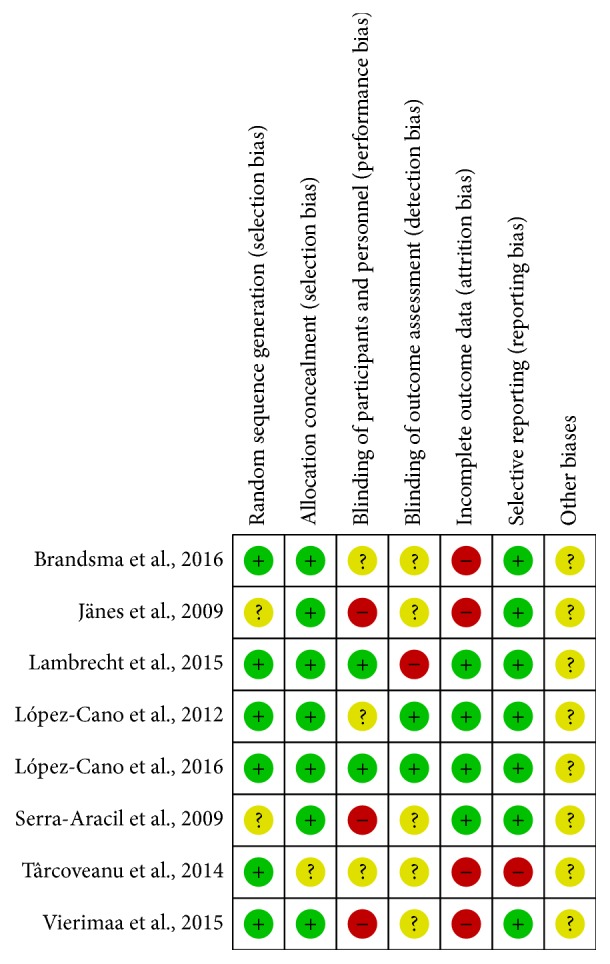
Risk of bias summary: author's judgement about risk of bias according to the Cochrane Risk of Bias Tool. +, high quality, −, low quality, and ?, unable to determine.

The basic characteristics of the RCTs are presented in Table 1. The 522 participants enrolled in the RCTs were divided into the following 2 groups: mesh group (underwent permanent colostomy with prophylactic mesh application) and control group (underwent conventional colostomy). The follow-up time ranged from 3 to 60 months. Patients lost to follow-up were taken into account in all studies, except the trial by Vierimaa et al. [27]; therefore, per protocol analysis was applied at the end. The results of the methodological quality of the included studies are presented in Figure 2.

### 3.2. Outcomes of the Pooled Studies

#### 3.2.1. Parastomal Hernia

**Figure 3:**
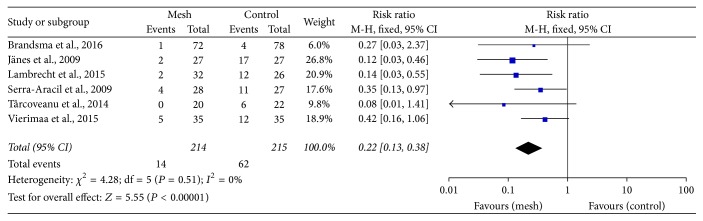
Forest plot for clinically detected parastomal hernia.

**Figure 4:**
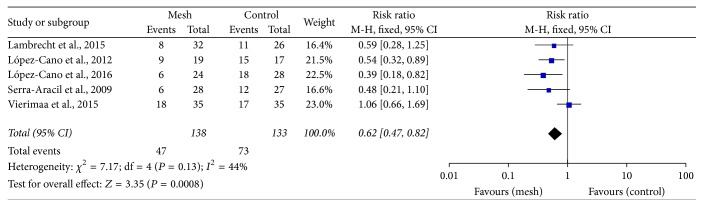
Forest plot for radiologically detected parastomal hernia.

All 8 RCTs reported the incidence of parastomal hernia. Three trials used clinical assessment [18, 20, 21, 24, 25], 2 trials used computed tomography (CT) to detect parastomal hernia [23, 28], and the remaining 3 trials used both methods [22, 26, 27]. Six trials showed that the rate of clinically detected parastomal hernia was lower in the mesh group than in the control group (RR: 0.22; 95% CI: 0.13–0.38; *P* < 0.00001) (Figure 3) [18, 20–22, 24–27]. Cingi et al. reported a higher rate of parastomal hernia when CT was used as the diagnostic tool than when clinical assessment was used [32]. Additionally, radiological method also favoured the prophylactic mesh as a positive technique in prevention of parastomal hernia (RR: 0.62; 95% CI: 0.47–0.82; *P* = 0.0008) (Figure 4). Accordingly, the diagnostic rate was higher with CT than with clinical assessment.

**Figure 5:**
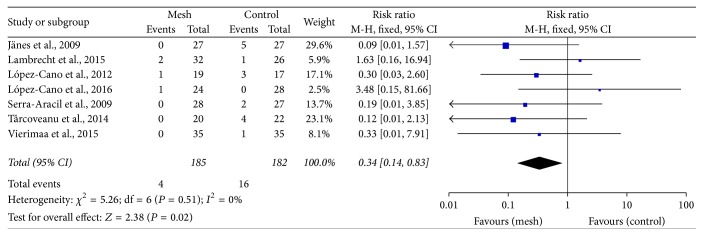
Forest plot for surgical repair for parastomal hernia.

Seven trials reported surgical repair for parastomal hernia [21–24, 26–28]. The surgical repair rate for parastomal hernia was lower in the mesh group than in the control group (RR: 0.34; 95% CI: 0.14–0.83; *P* = 0.02) (Figure 5).

#### 3.2.2. Infectious Complications

**Figure 6:**
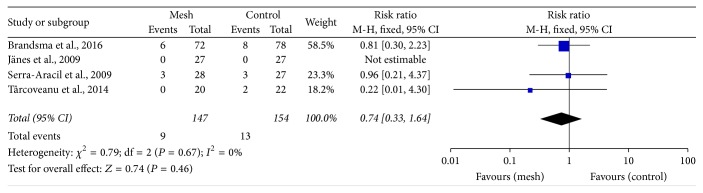
Forest plot for wound infection.

**Figure 7:**
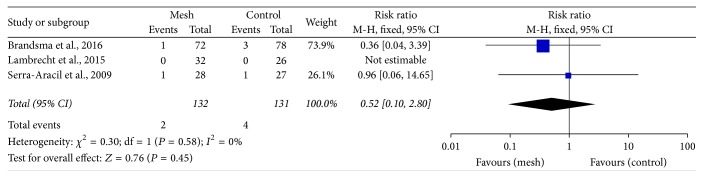
Forest plot for peristomal infection.

**Figure 8:**
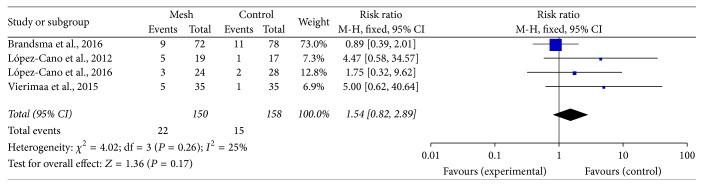
Forest plot for perineal wound infection.

Pooled data from the trials showed that applying a mesh at the time of fashioning the colostomy would not increase the risk of wound infection (RR: 0.74; 95% CI: 0.33–1.64; *P* = 0.46) (Figure 6) and peristomal infection (RR: 0.52; 95% CI: 0.10–2.80; *P* = 0.45) (Figure 7). Additionally, according to 4 trials, mesh application did not increase the incidence of infectious complications related to the mesh [21, 23, 25, 28]. The perineal wound infection rate was higher in the mesh group than in the control group; however, the difference was not significant (RR: 1.54; 95% CI: 0.82–2.89; *P* = 0.17) (Figure 8).

#### 3.2.3. Noninfectious Complications

**Figure 9:**
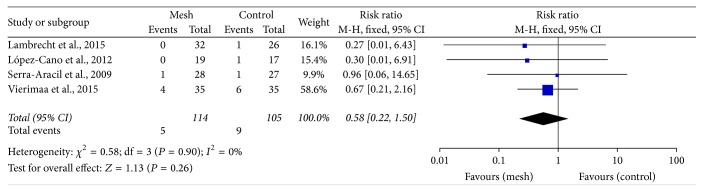
Forest plot for stomal necrosis.

**Figure 10:**
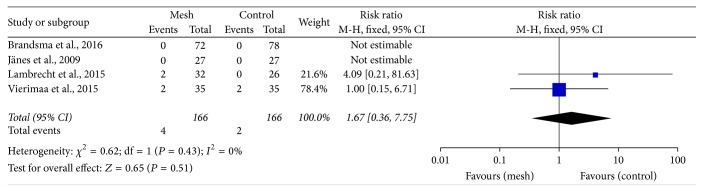
Forest plot for stomal stenosis.

There were no differences in the incidences of stomal necrosis (RR: 0.58; 95% CI: 0.22–1.50; *P* = 0.26) (Figure 9) and stomal stenosis (RR: 1.67; 95% CI: 0.36–7.75; *P* = 0.51) (Figure 10) between the mesh and control groups.

## 4. Discussion

Presently, patients who undergo colostomy seek a high quality of life. However, a large proportion of patients develop complications related to colostomy [33], and sometimes these complications can be life-threatening [34]. Among these complications, parastomal hernia is one of the major issues. One-third of patients who develop parastomal hernias may need operative treatment [35, 36]. Although techniques, such as mesh reinforcement and stoma repositioning, have been used to repair an affected abdominal wall, the results may be frustrating [12]. Therefore, the prevention of parastomal hernia is extremely important. Prophylactic mesh application at the time of stoma formation appears to help prevent herniation [37, 38]. Considering that parastomal hernia occurs frequently after colostomy [12, 39], our meta-analysis on the benefit of prophylactic mesh application after colostomy is important. Several systematic reviews have discussed whether prophylactic mesh application is beneficial [1, 40–42]; however, only 3 RCTs with small sample sizes were evaluated, both colostomy and ileostomy were considered, and mesh-related complications were not appropriately assessed. In our meta-analysis, 4 single-center and 4 multicenter studies with 522 participants were pooled to evaluate the value of the prophylactic mesh.

We found that, in clinical and radiological assessments, the occurrence of parastomal herniation reduced with prophylactic mesh application. Additionally, according to our pooled result, the operative treatment for parastomal hernia reduced with prophylactic mesh application. One trial in our meta-analysis reported that the stoma aperture was much smaller in the mesh group than in the control group, indicating that patients in the control group were susceptible to the development of parastomal hernia [26]. The follow-up time of the enrolled studies ranged from 3 to 60 months. Considering that the occurrence of parastomal hernia increases as the follow-up time is prolonged [43], a short follow-up time in the trial by Brandsma et al. might have caused a potential bias [25]. However, the occurrence of parastomal hernia remained low in the mesh group after elimination of this study (RR: 0.22; 95% CI: 0.13–0.37; *P* < 0.00001).

It appears that the use of CT may contribute to a high detection rate of parastomal hernia [4, 32], and this is consistent with our result (Figures 3 and 4). Janson et al. reported that prophylactic mesh application could reduce the rate of parastomal hernia formation in patients undergoing laparoscopic sigmoidostomy [44]. However, as this previous study was not a controlled trial, it could not be deduced whether the laparoscopic technique was superior to the open technique. In our meta-analysis, patients underwent laparoscopic surgeries with the placement of a mesh in an intraperitoneal/onlay position [23, 27, 28] and underwent open surgery in a sublay position [18, 21, 22, 24–26]. Considering that most patients underwent clinical examinations to detect parastomal hernia in the open surgery group and underwent CT in the laparoscopy group, we did not assess the most appropriate type of surgery or position of the mesh. Further trials are needed to compare different surgical procedures involving prophylactic mesh application.

Surgeons have been worried that mesh application close to the intestine may increase the risk of infection and cause complications, such as fistula and intestinal obstruction [45]. Our meta-analysis found that the rate of infection, including wound infection and peristomal infection, did not increase with mesh application (Figures 6 and 7). Four trials reported that no mesh-related infection occurred [21, 23, 25, 28]. The perineal infection rate tended to be higher in the mesh group than in the control group (without significance), and this might be associated with the abdominoperineal resection itself rather than with the existence of the mesh [46]. In addition, we noted that stomal necrosis and stenosis were not higher in the mesh group than in the control group (Figures 9 and 10). In 4 trials, fistula formation was not noted in the mesh group [21, 22, 24, 26]. Moreover, mesh application did not cause pain at the stomal site [25, 27]. Our pooled results are consistent with the findings of some non-RCTs that showed promising outcomes with mesh placement at the time of colostomy formation and absence of an increase in the complication rate [38, 47]. Lee et al. performed a cost effectiveness analysis and found that the cost was lower and the ability to prevent parastomal hernia was greater with prophylactic mesh application in patients who underwent permanent colostomy than with the conventional method [48].

In the included trials, surgeons used the keyhole technique, which involves an incision at the center of the mesh. The disadvantage of this approach is that the incision will enlarge after a long follow-up [23, 28]. Laparoscopy is developing rapidly and is being widely used; however, a new technique for mesh application is needed. A new method named Sugarbaker and its modifications have been widely applied in laparoscopy [49, 50]. The recurrence rate after parastomal hernia repair was found to be lower with the Sugarbaker technique than with the keyhole technique [51]. In a trial by López-Cano et al., this new technique showed promising results with regard to the prevention of parastomal hernia [28, 52]. Further RCTs should be performed to confirm the effectiveness of this new technique.

However, the present meta-analysis had some limitations. First, the sample size was not sufficiently large, and this might have resulted in bias. Second, we failed to evaluate the most optimal position, mesh material, and surgical type. Therefore, further trials with a larger sample size should be performed to ensure a reliable and powerful analysis. Finally, we included some trials with a short follow-up. Considering that the occurrence of parastomal hernia increases after 10 years [53], trials with a longer follow-up period are needed to confirm the results.

## 5. Conclusion

Our meta-analysis demonstrated that prophylactic mesh application at the time of primary colostomy formation is a promising method for the prevention of parastomal herniation. This approach might not completely prevent parastomal herniation but might reduce the incidence of parastomal herniation without increasing the incidence of complications in the long term. Therefore, it may be the preferred option in patients undergoing permanent colostomy.

## Figures and Tables

**Table 1:** Characteristics of included studies.

Author	Year	Center	Location	Sample size (M/C)	Type of surgery	Type of mesh	Location of mesh	Sex M : F M/C	Age M/C (year)	BMI M/C (kg/m^2^)	Follow-up (month)	Diagnosis of hernia
Jänes [18, 20, 21]	2004 2009	Single	Sweden	27/27	Open	Vypro mesh (polypropylene + polyglactin 910)	Sublay	15 : 12/16 : 11	70/71	26/27	60	Clinical exam

Serra-Aracil et al. [22]	2009	Single	Spain	28/27	Open	Ultrapro mesh (polypropylene + poliglecaprone 25 + 2 monofilament materials)	Sublay	19 : 5/16 : 8	67.5/67.2	25.6 : 27.3	29 (median)	Clinical exam + CT

López-Cano et al. [23]	2012	Single	Spain	19/17	Laparoscopic	PROCEED surgical mesh (polypropylene + polydioxanone)	Intraperitoneal/onlay	11 : 8/7 : 10	72.2/65.9	26.3 : 27.5	12	CT

Târcoveanu et al. [24]	2014	Single	Romania	20/22	Open	NA	Sublay	NA	NA	NA	20 (median)	Clinical exam

Brandsma et al. [25]	2016	Multicenter	Netherlands	72/78	Open	Parietene light mesh (monofilament polypropylene)	Sublay	43 : 29/49 : 29	63.6/63.1	26.7 : 26.5	3	Clinical exam

Lambrecht et al. [26]	2015	Multicenter	Norway	32/26	Open	ProLite Ultra mesh and parietene light mesh (polypropylene)	Sublay	22 : 10/21 : 5	64/63	24.6 : 25.5	40	Clinical exam + CT

Vierimaa et al. [27]	2015	Multicenter	Finland	37/38	Laparoscopic	DynaMesh-IPOM (polyvinylidene fluoride + polypropylene)	Intraperitoneal/onlay	18 : 17/19 : 16	67.1/65.1	26.2 : 25.4	12	Clinical exam + CT

López-Cano et al. [28]	2016	Multicenter	Spain	24/28	Laparoscopic	ETHICON mesh (poliglecaprone-25 film + polypropylene)	Intraperitoneal/onlay	21 : 3/16 : 8	70.5/67.3	25.3 : 26.9	26 (median)	CT

M/C: mesh group versus control group; M : F: male : female; sublay: between the rectus muscle and posterior rectus sheath; NA: not available; CT: computed tomography.
